# Prediction of Motor Recovery in the Upper Extremity for Repetitive Transcranial Magnetic Stimulation and Occupational Therapy Goal Setting in Patients With Chronic Stroke: A Retrospective Analysis of Prospectively Collected Data

**DOI:** 10.3389/fneur.2020.581186

**Published:** 2020-10-20

**Authors:** Toyohiro Hamaguchi, Naoki Yamada, Takuya Hada, Masahiro Abo

**Affiliations:** ^1^Department of Rehabilitation Medicine, The Jikei University School of Medicine, Tokyo, Japan; ^2^Department of Rehabilitation, Graduate School of Health Sciences, Saitama Prefectural University, Koshigaya, Japan

**Keywords:** transcranial magnetic stimulation, occupational therapy, stroke, motor paralysis, prediction

## Abstract

Recovery from motor paralysis is facilitated by affected patients' recognition of the need for and practice of their own exercise goals. Neurorehabilitation has been proposed and used for the treatment of motor paralysis in stroke, and its effect has been verified. If an expected score for the neurorehabilitation effect can be calculated using the Fugl-Meyer Motor Assessment (FMA), a global assessment index, before neurorehabilitation, such a score will be useful for optimizing the treatment application criteria and for setting a goal to enhance the treatment effect. Therefore, this study verified whether the responsiveness to a treatment method, the NovEl intervention using repetitive transcranial magnetic stimulation and occupational therapy (NEURO), in patients with post-stroke upper extremity (UE) motor paralysis could be predicted by the pretreatment FMA score. No control group was established in this study for NEURO treatment. To analyze the recovery of the motor function in the UE, delta-FMA was calculated from the pre- and post-FMA scores obtained during NEURO treatment. The probability of three levels of treatment responsiveness was evaluated in association with delta-FMA score (<5, 5 ≤ delta-FMA <10, and ≥10 as non-responders; responders; and hyper-responders, respectively) according to the reported minimal clinically important difference (MCID). The association of the initial FMA scores with post-FMA scores, from the status of the treatment responsiveness, was determined by multinomial logistic regression analysis. Finally, 1,254 patients with stroke, stratified by FMA scores were analyzed. About 45% of the patients who had FMA scores ranging from 30 to 40 before treatment showed improvement over the MCID by NEURO treatment (odds ratio = 0.93, 95% CI = 0.92–0.95). Furthermore, more than 25% of the patients with more severe initial values, ranging from 26 to 30, improved beyond the MCID calculated in the acute phase (odds ratio = 0.87, 95% CI = 0.85–0.89). These results suggest that the evaluated motor function score of the UE before NEURO treatment can be used to estimate the possibility of a patient recovering beyond MCID in the chronic phase. This study provided clinical data to estimate the effect of NEURO treatment by the pretreatment FMA-UE score.

## Introduction

Motor paralysis due to the aftereffects of stroke impairs the activities of daily living (ADL) and quality of life (QOL) of patients; it also affects their individual or social activities (1, 2). In particular, motor paralysis of the upper extremity has a large impact on ADL (3). Recovery from motor paralysis is facilitated by patients recognizing the need for and practicing their own exercise goals (4). The type of goals that patients set are related to their goal satisfaction scores, with impairment-based goals being rated significantly higher than activity-based and participation-based goals (5). It is known that patients' level of knowledge of their rehabilitation goals leads to effective treatment results (6). Thus, clinicians and patients are active partners in setting goals within stroke rehabilitation (5). In previous studies, some prognosis prediction systems were developed for motor paralysis (7–9), and they have been used to set goals for rehabilitation in patients with stroke.

Neurorehabilitation has been proposed and used for the treatment of motor paralysis in stroke, and its effect has been verified (10–14). One of the treatment methods, the NovEl intervention Using Repetitive transcranial magnetic stimulation and Occupational therapy (NEURO), facilitates peripheral muscle movement by controlling the excitability of the motor cortices by repetitive transcranial magnetic stimulation (rTMS). It also promotes peripheral muscle exercise and practice, for the active use of the paralyzed upper extremity (15, 16). NEURO's efficacy has been proved in a randomized controlled study (17). To date, many patients have been treated by using NEURO; however, the prediction regarding whether patients' recovery from motor paralysis after treatments can be predicted before treatment, has not been verified. If the Fugl-Meyer Motor Assessment (FMA) score before treatment can be used to predict NEURO treatment response, the score can be used as an effective goal for rehabilitation, by patients and therapists.

The minimal clinically important difference (MCID) of motor paralysis in the upper extremity has been investigated (18–20). If the expected value of an effect exceeding MCID can be calculated using FMA score measured before NEURO treatment, such a value will be useful for optimizing the treatment application criteria and setting a goal to enhance the treatment effect. For that purpose, it is sufficient to retroactively analyze the band of the FMA score before NEURO for a patient who is significantly improved. Therefore, this study verified whether the responsiveness of NEURO treatment for patients with post-stroke upper extremity motor paralysis could be predicted by the pre-treatment FMA score.

## Methods

### Participants

This is a multi-institutional open-label study without control patients. In January 2019, we surveyed the medical records of all patients with post-stroke muscle paralysis who had been admitted to six participating institutions (Jikei University Hospital, Jikei Third Hospital, Tokyo General Hospital, Kyoto Ohara Memorial Hospital, Nishi-Hiroshima Rehabilitation Hospital, Shimizu Hospital) between March 2010 and December 2018 for NEURO. For patients who had been treated with NEURO, the inclusion criteria were based on the TMS guidelines (21, 22) as follows: (1) upper limb hemiparesis categorized as cerebral infarction or cerebral hemorrhage; (2) age >20 years; (3) ≥4 months since stroke; (4) history of a single stroke only (no bilateral cerebrovascular lesions); (5) no cognitive deficits (a Mini Mental State Examination score ≥26); (6) no active physical or mental illness requiring medical management; (7) no history of convulsion for ≥1 year; (8) no intracranial metal clips or intracardiac pacemaker; and (9) no history of neurolytic nerve block (phenol or botulinum toxin) to the affected upper limb.

To verify if the upper extremity function was maintained after NEURO, patients were excluded: (1) if they did not have at least one FMA score before and after treatment, (2) if they had an initial FMA for upper extremity (FMA-UE) score <26/66, with severe motor impairment (15, 23), and those with a diagnosis of subarachnoid hemorrhage were excluded.

### NEURO and Occupational Therapy (OT) Sessions

OT was provided in addition to conducting NEURO sessions; therapy was planned to suit the needs of each patient. All the patients were hospitalized for 15 days to receive rTMS (15) and OT (24). During hospitalization, each patient received a 40-min rTMS session and an OT session every day, except on Sundays and the day of admission/discharge. All OT sessions were started within 10 min of rTMS.

Focal 1 Hz rTMS was applied to the contralesional hemisphere over the primary motor area, as described in previous studies (15, 23). A 70-mm figure–8 coil, attached to a MagPro R100 stimulator (MagVenture Company, Farum, Denmark) was used for rTMS application; for this, 2,400 pulses lasting for 40 min were applied. The stimulation intensity, set to 90% of the resting motor threshold for the first dorsal interosseous muscle on the unaffected side, was defined as the lowest intensity of the stimulation that could activate the motor-evoked potentials (MEP) of the muscle.

OT was performed twice daily, 6 days a week (excluding Sundays), and involved 60-min individual training sessions. The main goal of the OT sessions was to help the patients avoid focusing on the functional training and to encourage them to use their affected upper limbs again in daily activities. Treatment strategy included: (1) daily physical activities (e.g., eating), which included repetitive movements of the arm during flexion and extension; (2) individualized functional training tasks, which enabled the patients to improve on their movements, such as washing their hands and grasping small items with their paralyzed fingers; (3) elements involved in gross motor function, fine motor function, and multitasking; (4) clear demonstrations of the position of the upper limb to draw attention to this position during training; (5) staged interventions; (6) ADLs and unsupervised training tasks that could be continued after discharge; and (7) the provision of action feedback by passive intervention with verbal instructions.

### Sample Size Calculation for Analysis

Based on multivariate linear regression (*F*-tests), an effect size *f* ^2^ of 0.03, power (1—β) of 0.95, α of 0.05, and 6 explained predictors, the minimum sample size of each group was 674 patients (derived using G^*^Power 3.1) (25). Furthermore, with an expected dropout rate of 30%, we planned to recruit in total a minimum of 963 patients with stroke treated with NEURO. To examine whether detectable logistical separations in upper extremity motor function owing to NEURO could occur, about 1,000 patients with stroke were included in the analysis.

### Outcomes

**Figure 1:**
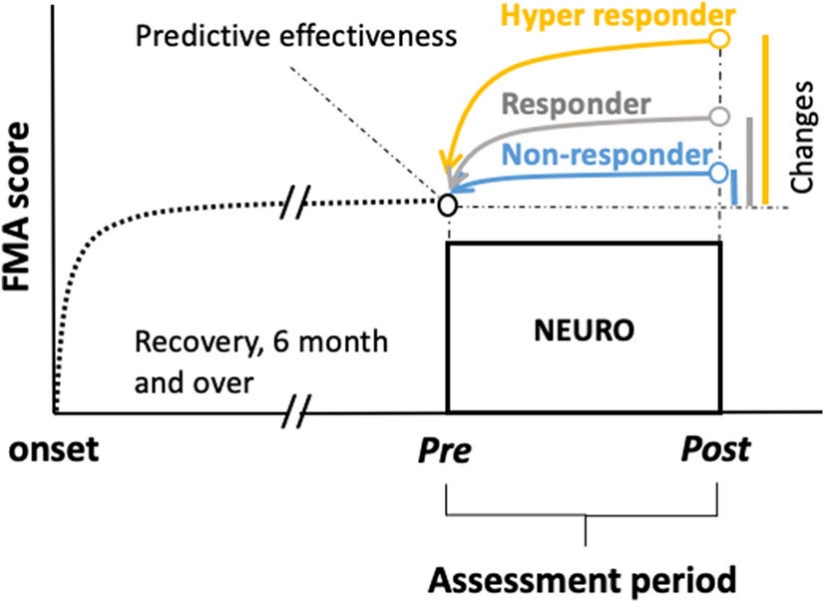
Chart showing schemes of retrospective prediction of the motor recovery of the upper extremities to determine the goals before treatment in patients with chronic stroke undergoing NEURO. To examine the hypothesis that being a responder, non-responder, or hyper-responder resulted in NEURO treatment can be discriminated using multinomial logistic regression to determine the association of FMA score between initial and delta scores in patients with post-stroke hemiparesis. Delta FMA-UE scores were calculated by subtracting the post- from the pre-NEURO score. The black dotted line drawn from the onset indicates the recovery curve from the acute to the chronic phase. The blue, gray, and yellow lines indicate the non-responders, responders, and hyper-responders of NEURO, respectively, regarding the recovery of motor function of the upper extremity. FMA-UE, Fugl-Meyer assessment of upper extremity; NEURO, NovEl intervention Using Repetitive transcranial magnetic stimulation and Occupational therapy.

The primary outcome was the FMA score. To predict the responsiveness to NEURO treatment from the initial score of FMA-UE, FMA scores (before and after treatment), age, sex, diagnosis (cerebral infarction or intracerebral hemorrhage), the dominant hand, and the time it took to recover motor function after the onset of stroke were investigated (Figure 1).

### Clinical Evaluation of the Motor Function

The motor function of the affected upper extremity was evaluated on both the day of the admission and discharge using FMA score. The FMA was devised in 1975 (26), and is a global assessment index used to quantitatively evaluate the recovery of post-stroke hemiparetic limbs. The FMA has high interrater and test-retest reliability, as described previously (27). The FMA is a performance-based quantitative measure made up of 33 items used to evaluate the upper limb motor function. Each item is rated on a 3-point ordinal scale (0 = cannot perform, 1 = can perform partially, and 2 = can perform fully), with a maximum score of 66 points. The severity of paralysis according to the FMA score is distributed as follows: ≤ 25, 26–45, and 46–66 for severe, moderate, and mild paralysis, respectively (28–30). The MCID of FMA for the upper extremity in a population of patients with stroke is 4–10 points in the acute or subacute phase (19, 20), and 5 points in the chronic phase (31).

### Statistical Analyses

To analyze the recovery of the motor function in the upper extremity, delta-FMA was calculated from the pre- and post-FMA scores obtained during NEURO treatment. In this study, the probability of the three levels of treatment responsiveness was evaluated in association with the delta-FMA score (<5, 5 ≤ delta-FMA <10, and ≥10 as non-responders; responders; and hyper-responders, respectively) according to previous studies (19, 20, 31). The association of the initial FMA scores with post-FMA scores, from the status of the treatment responsiveness, was determined by multinomial logistic regression analysis. The principle of multinomial logistic regression analysis requires that the probability (*p*) of the three levels (non-responders, responders, and hyper-responders) of the dependent variable, delta-FMA score, be fitted. The probability for the non-responders was the reference level; then the regression models were developed as follows:

$$\begin{array}{l} \text{g}\left({\text{x}}_{\;\text{nonresponders}}\right)\;=\;\frac{1}{1+{\text{e}}^{\text{f}\left({\text{x}}_{\;\text{responders}}\right)}\;+\;{\text{e}}^{\text{f}\left({\text{x}}_{\;\text{hyper}-\text{responders}}\right)}}\\ \;(1:\text{non-responders}) \end{array}$$

$$\begin{array}{l} f\left({\text{x}}_{\text{ responders}}\right)={\text{intercept}}_{\text{ responders | nonresponders }}\\ \;+\;\beta_{\text{ responders | nonresponders}}\;{\text{x}}_{\text{i}} \end{array}$$

$$\begin{array}{l} f\left({\text{x}}_{\text{ hyper-responders}}\right)={\text{intercept}}_{\text{ hyper-responders | nonresponders }}\\ \;+\;\beta_{\text{ hyper-responders | nonresponders }}{\text{x}}_{\text{i}} \end{array}$$

$$\begin{array}{l} \text{g}\left({\text{x}}_{\;\text{responders}}\right)\;=\;\frac{{\text{e}}^{\text{f}\left({\text{x}}_{\;\text{responders}}\right)}}{1\;+\;{\text{e}}^{\text{f}\left({\text{x}}_{\;\text{responders}}\right)}\;+\;{\text{e}}^{\text{f}\left({\text{x}}_{\;\text{hyper}-\text{responders}}\right)}}\\ \;(2:\text{responders}) \end{array}$$

$$\begin{array}{l} \text{g}\left({\text{x}}_{\;\text{hyper}-\text{responders}}\right)\;=\;\frac{{\text{e}}^{\text{f}\left({\text{x}}_{\;\text{hyper}-\text{responders}}\right)}}{1\;+\;{\text{e}}^{\text{f}\left({\text{x}}_{\;\text{responders}}\right)}\;+\;{\text{e}}^{\text{f}\left({\text{x}}_{\;\text{hyper}-\text{responders}}\right)}}\\ \;(3:\text{hyper-responders}) \end{array}$$

where **x**_**i**_, the initial-FMA-UE score, was the explanatory variable, **β**_**i**_ and **intercept**_**i**_ is the partial regression coefficient in each group, and **e** is Napier's constant. Therefore, for the multilevel responses, the cumulative probability was calculated at each level to generate a simple regression coefficient. The covariates influencing the recovery of the upper limb motor paralysis after treatment were: (1) age, (2) gender, (3) time from stroke onset to NEURO initiation, and 4) the dominant hand. To identify the model, the Akaike Information Criterion (AIC) was used (32). Applicability of the predictive model was assessed using McFadden's coefficient of determination, *R*^2^, between the initial score and the delta-FMA scores for all 1,254 patients (33). All statistical analyses were performed using R 3.6.0 software (R Foundation for Statistical Computing, Vienna, Austria).

## Results

**Figure 2:**
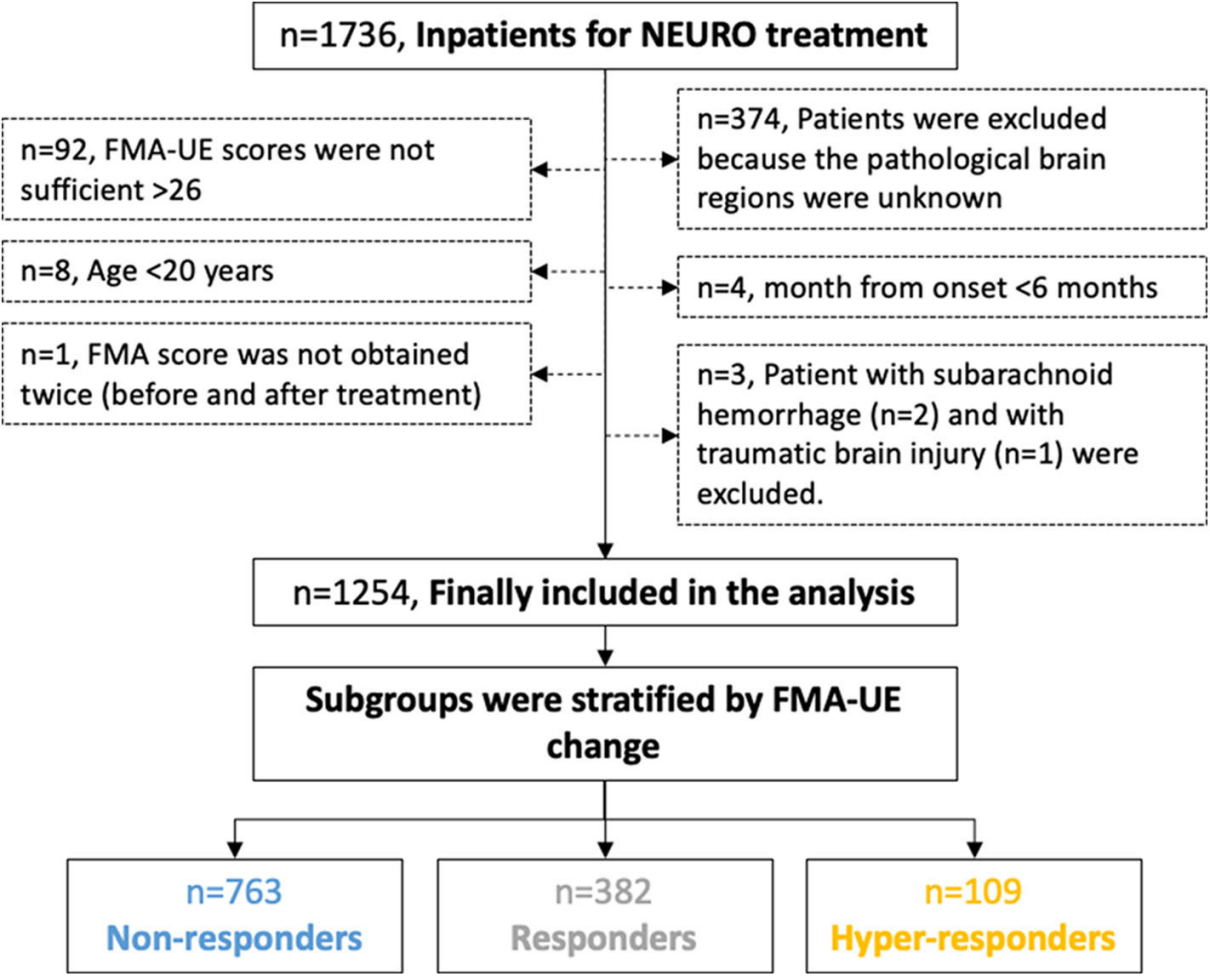
Flow chart of the study design protocol for the data acquisition and the selection of the participants for the analysis. The participants were divided into three groups by the reported MCID of FMA-UE. MCID, minimal clinically important difference; FMA-UE, Fugl-Meyer assessment of upper extremity; NEURO, NovEl intervention Using Repetitive transcranial magnetic stimulation and Occupational therapy.

Figure 2 shows the flow chart of the study design and patients selection based on the diagnosis. The median age and interquartile range of all patients were 63 and 56–70 years respectively. Table 1 summarizes the clinical characteristics of the patients; the distributions of the characteristics were comparable across groups. Right-handed patients accounted for 95%, which is approximately equal to the same proportion for all Japanese. There were about twice as many males as females.

**Table 1:** Patient characteristics among groups at baseline.

**Characteristic**	**Non-responders**	**Responders**	**Hyper-responders**
Participants (n)	763 (61%)	382 (26%)	109 (13%)
Age (years)	63 (56–70)	63 (55–70)	64 (56–69)
Sex (n)
Female	247 (32%)	123 (32%)	41 (38%)
Male	516 (68%)	259 (68%)	68 (62%)
Paralysis side (n)
Left	315 (41%)	173 (45%)	55 (50%)
Right	448 (59%)	209 (55%)	54 (50%)
Dominant hand (n)
Left	35 (5%)	23 (6%)	5 (5%)
Right	728 (95%)	359 (94%)	104 (95%)
Diagnosis
CI	387 (51%)	194 (51%)	51 (47%)
ICH	376 (49%)	188 (49%)	58 (53%)
Time from onset (months)	41 (23–74)	41 (24–75)	37 (21–58)
FMA-UE (in charge)	54 (46–60)	47 (39–52)	40 (33–45)

*Values are n (%) or median (interquartile range). CI, cerebral infarction; ICH, intracranial hemorrhage; FMA-UE, Fugl-Meyer Assessment score*.

**Figure 3:**
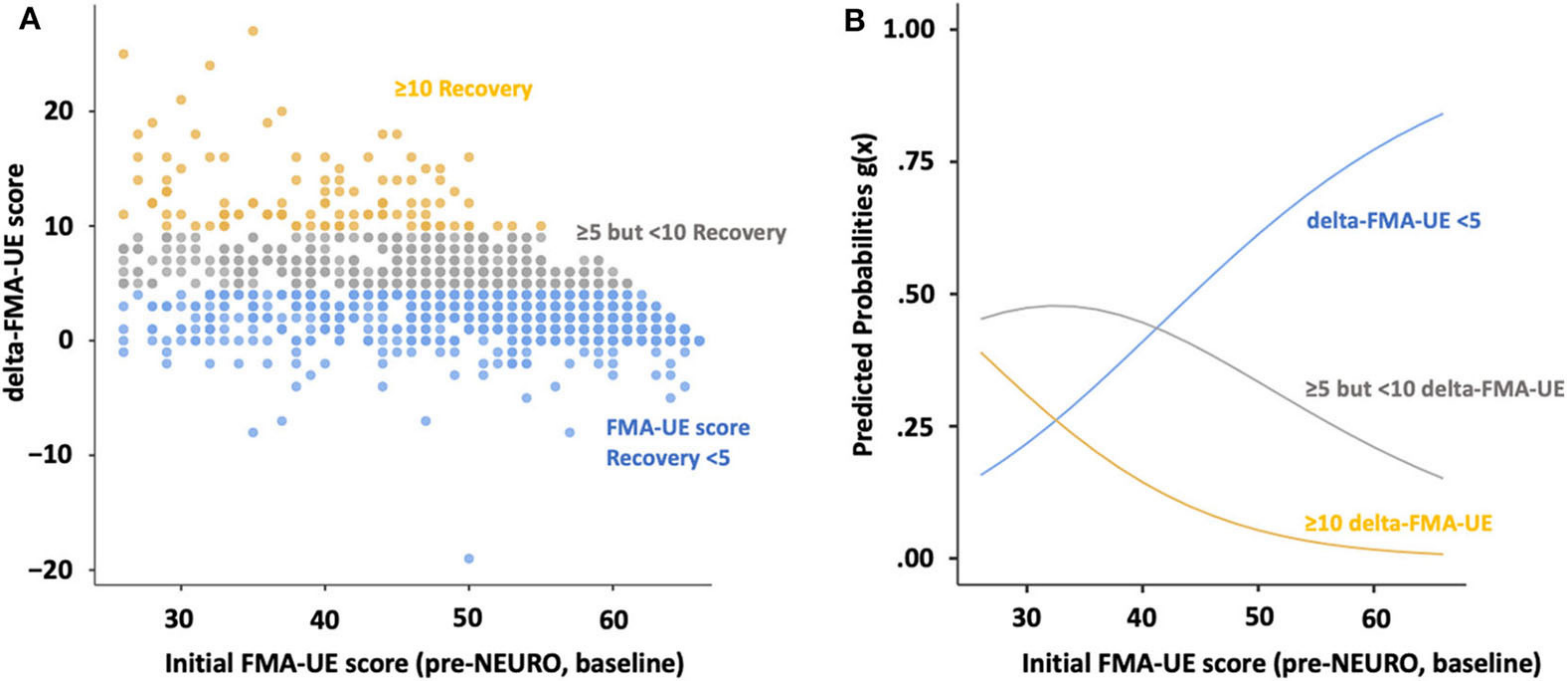
Scatterplots and multinomial logistic probability plots showing the association between level of agreement for initial- and delta FMA score. (A) Initial FMA-UE score plots and histogram of FMA-UE score change for the upper extremities are divided by recovery, according to MCIDs. (B) The logistic curves were discriminated by the probability of being non-responders (delta-FMA-UE score <5 points, blue line), responders (5 ≤ delta-FMA-UE, gray line <10 delta-FMA-UE), and hyper-responders (delta-FMA-UE, yellow line ≥10). FMA: Fugl–Meyer assessment; NEURO, NovEl intervention Using Repetitive transcranial magnetic stimulation and Occupational therapy; MCID, minimal clinically important difference.

The multinomial logistic regression model fitted showed statistically significant valid logistic probability between delta- and the initial FMA score, adjusted for covariates, age, sex, time from onset, diagnosis, and dominant hand (McFadden's *R*^2^ = 0.103, AIC = 1,999, χ^2^ = 227, *p* < 0.001) (Table 2). Time-series plots of the FMA scores are shown in Figure 3. The logistic curves discriminating between the probability of being responders (5 ≤ delta-FMA <10) from non-responders (delta-FMA <5) showed a significant model fit (*z* = 5.31; *p* < 0.001; odds ratio = 15.5, 95% Cl = 5.7–42.9). Similarly, hyper-responders (delta-FMA ≥10) and non-responders (delta-FMA <5) were differentiated according to the initial-FMA score (*z* = 6.38; *p* < 0.001; odds ratio = 166.8, 95% Cl = 34.6–803.5).

**Table 2:** Model coefficients of treatment responsiveness and initial FMA-UE score.

**Separated responsiveness**	**Predictor**	**Estimate**	**95% CI**	***z***	***p***
Responders|Non-responders	Intercept	2.75	1.73, 3.76	5.31	<0.001
	Initial FMA-UE	−0.07	−0.08, −0.06	−10.01	<0.001
	Age	−0.00	−0.01, 0.01	−0.17	0.863
	Sex	0.02	−0.25, 0.30	0.16	0.871
	Month from onset	−0.00	−0.00, 0.00	−0.25	0.806
	Diagnosis	0.04	−0.22, 0.30	0.30	0.764
	Handedness	0.35	−0.21, 0.92	1.22	0.224
Hyper-responders|Non-responders	Intercept	5.11	3.54, 6.69	6.38	<0.001
	Initial FMA-UE	−0.14	−0.16, −0.12	−11.64	<0.001
	Age	−0.01	−0.03, 0.01	−1.03	0.302
	Sex	−0.24	−0.69, 0.22	−1.02	0.306
	Month from onset	−0.00	−0.01, −0.00	−0.74	0.458
	Diagnosis	0.28	−0.16, 0.72	1.25	0.213
	Handedness	0.18	−0.85, 1.2	0.34	0.729

*FMA-UE, motor function score of upper extremity by Fugl-Meyer Assessment; N-R, non-responders; R, responders; H-R, hyper-responders*.

According to the multinomial logistic regression models, the probability of being a non-responders was 59.2% when the initial FMA score was 48.9. Similarly, when the initial FMA score was 38.8, the incidence of responders and hyper-responders was 45.5 and 16.0%, respectively (Table 3).

**Table 3:** Estimated marginal means of Fugl-Meyer Assessment score in upper extremity, compared with responsiveness of treatment.

**Initial** **FMA-UE**				**95% Confidence Interval**	
	**Responsiveness**	**Probability**	**SE**	**Lower**	**Upper**
38.8^−^	N-R	0.384	0.036	0.307	0.462
	R	0.455	0.039	0.372	0.539
	H-R	0.160	0.034	0.087	0.234
48.9^μ^	N-R	0.592	0.035	0.518	0.667
	R	0.347	0.033	0.277	0.419
	H-R	0.060	0.016	0.027	0.093
59.0^+^	N-R	0.760	0.030	0.695	0.825
	R	0.221	0.029	0.158	0.284
	H-R	0.019	0.006	0.005	0.032

*CI, cerebral infarction; ICH, intracerebral hemorrhage, N-R, nonresponders; R, responders; H-R, hyper-responders. –, mean – 1SD; μ, mean; +, mean + 1SD; FMA-UE, Fugl-Meyer Assessment score; SD, standard deviation; SE, standard error*.

## Discussion

Recently, the maximum recovery state of motor function of the upper extremity in patients with stroke hemiparesis, including spontaneous recovery, has been estimated, based on the measured acute phase value (7, 34–36). Subsequent studies have also shown that NEURO treatment may restore motor function in the upper extremities during the chronic phase (15, 17). In this study, motor function of the upper extremities, based on values measured prior to NEURO treatment, was used to estimate post-treatment recovery rates based on previously reported acute and chronic MCID levels (19, 20, 31). The results of this study showed that about 45% of patients in the chronic stage who had FMA scores ranging from 30 to 40 before treatment showed improvement over the MCID by NEURO treatment. Furthermore, more than 25% of the patients with more severe initial values ranging from 26 to 30 improved beyond the MCID calculated in the acute phase. These results suggest that the evaluated motor function scores of the upper extremities before NEURO treatment can be used to estimate the occurrence of patients recovering beyond MCID among the patients in the chronic phase.

It is known that the effect of rehabilitation is enhanced when patients recognize the need to achieve their own goals and actively engage in pursuing them (37, 38). In addition, patients who practice self-efficacy affect the recovery of the upper limb motor function (4). Patients' recognition of the need to have their own behavioral goals and practice upper limb exercises display enhanced performance (4). Therefore, prediction of the treatment effect on the patient is important for the therapist and can facilitate patients' consent and cooperation with the treatment (39). To judge from the results of this study, the extent of recovery by NEURO treatment can be predicted, to some extent, from the patients' pre-treatment upper extremity functional evaluation, and this is useful information for the attending physician to provide the patient.

In this NEURO treatment, low frequency (LF)-rTMS was used. Ferbert et al. discovered that stimulation of the contralateral motor cortex immediately after stimulation of the motor area reduces the potential of stimulation of the contralateral hemisphere to evoke finger muscles (40). Moreover, Wards et al. reported that in the case of unilateral brain injury, the activity of the contralateral hemisphere was increased, and hyperactivity of the non-lesional hemisphere excessively induced the interhemispheric inhibition on the lesional side (41). In other words, unbalanced excitement of the cerebrum on the non-lesioned hemisphere adversely affects functional improvement. Since nervous activity is suppressed by LF-rTMS, the activity of the non-lesional hemisphere can be suppressed by applying LF-rTMS to the motor cortex of the non-lesional hemisphere (42), and suppression of interhemispheric inhibition of the non-lesional hemisphere indirectly increases the activity of the lesional side (43). On the other hand, high-frequency (HF)-rTMS evokes nervous activity and stimulates the motor cortex of the lesional hemisphere to enhance activity at the lesional site directly (44). Intensive upper-limb exercises are performed immediately after rTMS while the neurological activity of patients with stroke is adjusted, thus facilitating motor function (10, 45). The stimulation method corresponding to the effects of the neuromodulation in patients with various levels of disability will hopefully be of use in the clinical setting after further validation of its effectiveness.

In this study, recovery from motor paralysis in the upper extremities with NEURO treatment tended to occur more frequently in patients with moderate paralysis. In the chronic phase of stroke, the most widely accepted explanation for the efficacy of the 1-Hz stimulation of the unaffected hemisphere is the reduction in the abnormally high transcallosal inhibition toward the affected hemisphere (46, 47). In the acute phase, Wang et al. reported that HF-rTMS and exercise therapy could improve motor recovery at about a 10 FMA-UE score in patients with severe hemiplegic stroke (48). Similarly, Watanabe *et al*. reported that patients in the acute phase had reduced muscle spasticity and recovery of motor function with rTMS (49). Even when motor paralysis was severe, improvement of motor function in the upper extremities was observed by adjusting the excitability of the motor cortex in this study. In addition, the FMA-UE assesses the patients, post-stroke, per the sequential recovery stages (26). The FMA items are hierarchically organized from synergistic to voluntary movements. Synergistic movements exhibit abnormally stereotyped behavior that does not allow the combination of different movement patterns. For example, an attempt to raise the arm results in elbow flexion, shoulder abduction, and internal rotation. The flexor and extensor synergy components were tested before the movements combining the synergies with the movements out of synergy. Ya-yun et al. reported that the increase in FMA-UE score reflects the improvement of the proximal upper extremity movement (50). It is considered that the rTMS treatment improved the FMA score, and the patients with more severe motor paralysis had improved proximal upper limb movements. Schambra *et al*. reported that there was no difference in FME-UE score recovery with or without MEP in patients in the acute phase, but there was less improvement in patients with high FMA scores than in those with low FMA scores, and FMA recovery curves plateaued below the reported normal levels for both the arm and hand (51). The lower response of patients with high motor function compared to moderately paretic patients in our study might be because the treatment-recovery values were low in patients with high motor function. Furthermore, Veldema et al. reported that in patients with stroke, severe hand dysfunction was associated with a strong suppression of the ipsilesional cortico-spinal excitability and a shift in excitability toward the contralesional hemisphere (52). In the same study, mild hand movement impairment was associated with a shift in cortico-spinal excitability toward the ipsilesional hemisphere. Therefore, ipsilesional HF-rTMS may be effective in mild paralysis. As the upper extremities become more active, patients may be willing to actively use it. The results of this study clinically suggested that even in more affected moderate cases of motor paralysis in the chronic phase, the effect of rehabilitation can be obtained in about 20% of patients, as in the acute phase.

Clinically (although not shown by the data in this study) and frequently, after the treatment there are highly psychologically satisfied patients, because they could use their own extremities and hands due to decreased finger clawing and because objects could be held by the paralyzed hands, even if the FMA score did not significantly change. Therefore, clinicians are required to explain to the patients how much they can improve and motivate them to participate in the treatment. To this end, further research should be conducted on the relationship between patients' motor function and their level of satisfaction, as well as the evaluation of gross and fine movement improvements.

There were some limitations to this study. Although the study did not include treatment data other than for NEURO, the patients included in the analysis may have received other treatments simultaneously, such as exercise therapy or OT. In addition, since the upper extremities are often used in ADL, the amount of functional recovery of the upper extremities is generally increased. The effects of the difference on non-NEURO treatments can be identified by comparing the recovery prediction accuracy between a non-NEURO-treated group and others treated with NEURO. There were more than 1,200 subjects in this study, and performing the stratified analysis described above requires larger samples.

## Conclusion

This study provided clinical data to estimate the effect of NEURO treatment by pre-treatment FMA-UE score. Further verification is required regarding the need for both the patients and therapists to undergo rehabilitation with the goal of recovery before and after treatment, which has a favorable effect on treatment outcomes.

## Data Availability Statement

The raw data supporting the conclusions of this article will be made available by the authors, without undue reservation.

## Ethics Statement

The studies involving human participants were reviewed and approved by The ethics committee of the Tokyo Jikei University School of Medicine and included an opt-out consent method (No. 20-041-5231). The patients/participants provided their written informed consent to participate in this study.

## Author Contributions

THam: analysis of data, data interpretation, and writing of the manuscript. NY: data interpretation and revisions. THad: data interpretation and revisions. MA: conception/design of the study, acquisition and analysis of data, data interpretation, writing of the manuscript, and revisions. All authors approved the submitted version and have agreed both to be personally accountable for the authors' contributions and to ensure that questions related to the accuracy or integrity of any part of the work, even ones in which the author was not personally involved, have been appropriately investigated, resolved, and the resolution documented in the literature.

### Conflict of Interest

The authors declare that the research was conducted in the absence of any commercial or financial relationships that could be construed as a potential conflict of interest.

## Abbreviations

ADL: activities of daily living
AIC: Akaike Information Criterion
FMA: Fugl-Meyer Motor Assessment
HF: high frequency
LF: low frequency
MCID: minimal clinically important difference
NEURO: NovEl intervention Using Repetitive transcranial magnetic stimulation and Occupational therapy
OT: occupational therapy
QOL: quality of life
rTMS: repetitive transcranial magnetic stimulation
SD: standard deviation
TMS: transcranial magnetic stimulation
UE: upper extremity.
